# Second language identity negotiation and willingness to communicate: the roles of belonging and cross-cultural contact

**DOI:** 10.3389/fpsyg.2026.1982398

**Published:** 2026-10-19

**Authors:** Lin Liang, Tingting Qiu

**Affiliations:** 1 Henan University of Science and Technology, Luoyang, China; 2 Jiangsu Normal University, Xuzhou, China

**Keywords:** cross-cultural contact quality, moderated mediation, second language identity negotiation, sense of belonging, willingness to communicate

## Abstract

**Introduction:**

Although both second language acquisition and cross-cultural psychology have increasingly recognised the importance of learners’ identity and intercultural experiences, the psychological mechanisms linking second language (L2) identity negotiation to willingness to communicate (WTC) remain insufficiently understood. Drawing on identity theory and the multi-level model of WTC, this study examined whether sense of belonging mediates the association between L2 identity negotiation and WTC and whether this indirect association varies according to the quality of intercultural contact.

**Methods:**

Using cluster sampling, questionnaire data were collected from 468 undergraduate students majoring in Spanish at Chinese universities. A moderated mediation model was tested to examine the mediating role of sense of belonging and the moderating role of intercultural contact quality.

**Results:**

The findings indicated that (1) L2 identity negotiation was positively associated with both sense of belonging and willingness to communicate; (2) sense of belonging partially mediated the association between L2 identity negotiation and willingness to communicate; and (3) intercultural contact quality moderated the first stage of the mediation process, such that the positive association between L2 identity negotiation and sense of belonging was stronger among learners reporting higher-quality intercultural contact.

**Discussion:**

These findings suggest that sense of belonging represents an important psychological mechanism linking L2 identity negotiation to willingness to communicate and that the quality of intercultural contact strengthens this association. The study extends existing research by integrating identity theory, the WTC framework, and intergroup contact theory, and provides empirical support for identity-oriented and intercultural approaches to Spanish language teaching.

## 1 Introduction

Driven by globalisation, the number of people learning Spanish, the world’s second most widely spoken native language, continues to increase. Over the past two decades, Spanish language programmes in Chinese universities have expanded rapidly, with growing numbers of students viewing Spanish proficiency as an important means of engaging with Spanish-speaking countries in cultural, economic, commercial, and academic contexts. However, language learning extends far beyond the acquisition of vocabulary, grammar, and communicative skills. It also involves learners’ identity development and motivational processes, raising questions such as Who am I? and Am I willing to communicate?

Willingness to communicate (WTC) is widely recognised as a central construct in second language acquisition because it reflects learners’ readiness to engage in communication in the target language. MacIntyre et al. (1998) conceptualised WTC in their pyramid model as a learner’s intention to use the second language for communication at a particular moment. According to this model, WTC is shaped not only by immediate situational influences but also by relatively stable individual characteristics, such as motivation, anxiety, and self-perceptions. Subsequent research has further demonstrated the dynamic and context-sensitive nature of WTC, showing that learners’ readiness to communicate can fluctuate considerably across situations and over short periods of time (Kang, 2005; MacIntyre and Legatto, 2011; Pawlak and Mystkowska-Wiertelak, 2015; Zhang et al., 2018). Previous research has also shown that WTC is positively associated with second language use and communicative engagement (Yashima, 2002; Yashima et al., 2004; Peng and Woodrow, 2010). It has therefore become an important construct for understanding learners’ communicative behaviour both inside and outside the classroom.

Despite the growing body of research on WTC, relatively little attention has been paid to how learners’ second language identity negotiation is associated with their willingness to communicate through emotional processes, particularly sense of belonging. Identity theory (Norton, 2000, 2013) conceptualises language learning as a process of continuous investment in imagined communities, during which learners negotiate their positions and identities within the target language community. This perspective is consistent with broader work linking language learning motivation, self-concept, and identity development (Dörnyei, 2005, 2009; Ushioda, 2011). Learners who successfully integrate their second language identity into their self-concept are more likely to experience positive emotional engagement with the target language community, which may in turn be associated with greater willingness to communicate.

Research in cross-cultural psychology has likewise demonstrated that the quality of contact with members of different cultural groups, rather than the frequency of such contact alone, is closely associated with attitudes, emotions, and behavioural intentions (Allport, 1954; Pettigrew, 1998; Pettigrew and Tropp, 2006). For learners of Spanish, cross-cultural contact quality may strengthen or weaken the association between second language identity negotiation and learners’ emotional experiences and communicative behaviour. Nevertheless, previous studies have tended to examine identity, sense of belonging, or contact separately, with relatively few attempts to integrate these factors into a single theoretical framework for understanding the relationships among identity negotiation, emotional processes, and communicative behaviour.

Based on this context, this study examines the correlations among second language identity negotiation, sense of belonging, willingness to communicate, and cross-cultural contact quality among undergraduate Spanish majors in Chinese universities. In particular, it investigates whether sense of belonging mediates the association between second language identity negotiation and willingness to communicate and whether cross-cultural contact quality moderates the association between second language identity negotiation and sense of belonging. The current study integrates three theoretical perspectives—identity theory, the WTC framework, and intergroup contact theory—to contribute to understanding the psychological and contextual mechanisms associated with willingness to communicate in L2 learning. It also offers empirical evidence that may inform identity-oriented pedagogy and the design of meaningful cross-cultural Spanish-language learning experiences.

## 2 Literature review and theoretical framework

### 2.1 Second language identity negotiation

Identity refers to an individual’s dynamic understanding of who they are within a particular social context. In second language acquisition research, the concept of identity is grounded in sociocultural perspectives. Norton (2000, 2013) conceptualised language learner identity as a dynamic and negotiated social position embedded in power relations. Rather than passively accepting inclusion or exclusion by the target language community, learners seek legitimate participation in imagined communities through continuous investment and identity negotiation. This process involves ongoing reflection on and reconstruction of their language competence, cultural orientation, and group affiliation.

At the empirical level, second language identity negotiation refers to the extent to which learners actively construct, adjust, and integrate their second language identity with their first language identity while using the target language (Block, 2007; Darvin and Norton, 2015). Longitudinal evidence has demonstrated that language learning and identity development are closely intertwined and that learners’ identities may change as their participation in the target language community develops (Gao et al., 2015). Previous research has also shown that successful identity negotiation is positively associated with learning engagement and learners’ motivation to use the target language (Gao, 2021; Papi and Hiver, 2020). More broadly, motivational research suggests that learners’ self-concepts, imagined future selves, and autonomous engagement play important roles in sustained language-learning behaviour (Dörnyei, 2005, 2009; Kormos and Csizér, 2014; Ushioda, 2011). Learners who are able to express an authentic sense of self through the second language and perceive their identities to be recognised by the target language community are generally more willing to participate in language learning activities From a self-determination perspective, experiences of autonomy, competence, and relatedness may also support more self-determined forms of language learning motivation (Ryan and Deci, 2000; Noels et al., 2019). These findings provide a useful basis for understanding how identity negotiation may be associated with subsequent emotional experiences and communicative behaviour. Evidence from Chinese learners of Spanish also suggests that changes in identity are closely related to learning motivation. Recent research has likewise shown that Chinese university students’ plurilingual and pluricultural orientations are closely associated with the development of language identity and intercultural engagement (Tang and Calafato, 2022). Learners who experience identity reinforcement or identity expansion through engagement with the target language and culture tend to report higher levels of self-confidence and learning engagement (Luo and García Parejo, 2022; Peng and Wu, 2022). From the perspective of identity theory, successful identity negotiation not only facilitates learners’ engagement with the target language but also strengthens their perceived psychological connection to the target language community, thereby providing a theoretical basis for the development of a sense of belonging. These findings reinforce the view that second language identity negotiation should be understood as a dynamic process through which learners continuously reconstruct their identities while engaging with diverse linguistic and cultural communities.

### 2.2 Sense of belonging

A sense of belonging refers to the subjective experience of feeling accepted, valued, and connected within a group (Baumeister and Leary, 1995). In educational psychology, it has consistently been identified as an important emotional factor associated with academic engagement, persistence, and wellbeing (Goodenow, 1993; Walton and Cohen, 2007). Within second language learning contexts, sense of belonging reflects learners’ perceived integration into both the language classroom community and the broader target language community.

Identity and sense of belonging are closely connected from a theoretical perspective. Learners who successfully negotiate their second language identity and perceive greater alignment between their self-concept and the target language community are more likely to experience a stronger sense of belonging (Block, 2007). Conversely, identity conflict or unsuccessful identity negotiation may be associated with feelings of alienation, thereby weakening learners’ perceived belonging within the language community. Accordingly, as an affective representation of learners’ perceived membership in the target language community, a sense of belonging is expected to function as an important psychological mechanism linking identity negotiation to subsequent communicative behaviour. Second language identity negotiation has been regarded as an important psychological antecedent of sense of belonging.

### 2.3 Willingness to communicate

Willingness to communicate (WTC) was originally introduced by McCroskey and Baer (1985) in research on first language communication and was subsequently extended to second language acquisition by MacIntyre et al. (1998), who proposed the well-known pyramid model of WTC. The model conceptualises WTC as the outcome of six interrelated layers of variables, ranging from relatively stable individual characteristics and intergroup climate to immediate communicative intentions. Subsequent research has demonstrated that WTC is a dynamic construct that varies according to situational, interpersonal, motivational, and affective conditions (Kang, 2005; MacIntyre and Legatto, 2011; Pawlak and Mystkowska-Wiertelak, 2015; Zhang et al., 2018). Building on this framework, Peng and Woodrow (2010) and Wen and Clément (2003) operationalised WTC as learners’ intentions to communicate both inside and outside the classroom, making it one of the most widely used outcome variables in second language research. Research has further demonstrated that learners’ motivational orientations, attitudes, perceived competence, and social-psychological characteristics are important correlates of WTC (Peng, 2022; Wei and Xu, 2022; Yashima et al., 2004). Classroom interaction can also shape learners’ moment-to-moment willingness to communicate, with teacher behaviour constituting an important contextual influence (Zarrinabadi, 2014). More recently, WTC has been examined across both classroom and extramural digital environments, highlighting the relationship between learners’ motivational self systems and their readiness to communicate across different contexts (Lee and Lu, 2023).

A substantial body of research suggests that affective and motivational factors are closely associated with WTC. In particular, learners’ sense of belonging within the language community has been identified as potentially important for communicative engagement (Cao, 2014; Khajavy et al., 2016). Meta-analytic evidence has likewise shown that positive emotions, including foreign language enjoyment, are associated with more adaptive language-learning outcomes (Botes et al., 2022). Learners who feel accepted within the language community and perceive their participation as valued may therefore be more willing to initiate communication. Together, these findings provide empirical and theoretical support for the proposed association between sense of belonging and willingness to communicate.

Within MacIntyre et al.'s (1998) multi-level model, sense of belonging can be understood as part of the broader social and affective context that facilitates learners’ readiness to engage in communication, thereby providing a theoretical rationale for positioning belonging as the mediator in the present study.

### 2.4 Cross-cultural contact quality

Intergroup contact theory (Allport, 1954) proposes that contact between members of different groups is more likely to promote positive intergroup outcomes when conditions such as equal status, shared goals, cooperation, and institutional support are present. Pettigrew (1998) further elaborated the processes through which intergroup contact may improve intergroup relations, while Pettigrew and Tropp (2006) demonstrated through meta-analysis that intergroup contact is consistently associated with more positive attitudes and behavioural intentions. Emotional processes play an important role in these associations. More recent studies have increasingly emphasised that the quality of contact may be more important than its frequency. High-quality contact characterised by equality, mutual respect, and meaningful interaction is more consistently associated with positive emotional and behavioural outcomes than contact that is merely frequent (Vezzali et al., 2015). Research on cross-cultural encounters has similarly highlighted the importance of meaningful interaction for language use and intercultural engagement (Tsang, 2022).

In second language learning, cross-cultural contact quality refers to the extent to which learners engage in authentic, equal, and positive interactions with native Spanish speakers and Spanish-speaking communities. Previous research suggests that cross-cultural experiences may be associated with cultural adaptation, identity development, and communicative confidence (Peng, 2015; Dewaele and MacIntyre, 2014; Tsang, 2022). Recent studies involving learners of languages other than English (LOTEs) have also highlighted that authentic language use in both digital environments and classroom settings is closely related to learners’ motivation and emotional experiences (Dewaele and Saito, 2024; Peng and Wu, 2022).

Nevertheless, the moderating role of cross-cultural contact quality within the proposed identity–emotion–behaviour framework has received relatively limited empirical attention. Therefore, cross-cultural contact quality is conceptualised as a contextual condition that may strengthen or weaken the extent to which successful identity negotiation translates into learners’ emotional integration with the target language community.

### 2.5 Theoretical model and research hypotheses

Based on the literature reviewed above, this study proposes a moderated mediation model to examine the relationships among second language identity negotiation, sense of belonging, willingness to communicate (WTC), and cross-cultural contact quality. Identity theory and investment theory suggest that second language identity negotiation is closely associated with learners’ emotional connection to the target language community. Although identity theory, the WTC framework, and intergroup contact theory originate from different research traditions, they provide complementary explanations for how second language learners transform identity-related experiences into communicative behaviour. Identity theory explains how learners negotiate their self-concept in relation to the target language community. This process is expected to foster a stronger sense of belonging, representing learners’ emotional integration into that community. The WTC model further suggests that such affective experiences constitute an important antecedent of learners’ willingness to communicate. Finally, intergroup contact theory specifies the contextual conditions under which identity negotiation is more likely to translate into belonging, highlighting the facilitating role of high-quality cross-cultural contact. Therefore, integrating these perspectives provides a coherent theoretical explanation for the proposed identity–belonging–WTC pathway.

This study also proposes that cross-cultural contact quality moderates the association between second language identity negotiation and sense of belonging. According to intergroup contact theory, positive and meaningful cross-cultural interactions provide opportunities for learners to validate and reinforce their identities within the target language community. Under conditions of higher cross-cultural contact quality, successful identity negotiation is more likely to be accompanied by a stronger sense of belonging. In contrast, when cross-cultural contact quality is relatively low, the association between identity negotiation and sense of belonging may be weaker because learners have fewer opportunities to develop meaningful emotional connections with the target language community. Therefore, this study focuses on the moderating role of cross-cultural contact quality in the first stage of the proposed mediation model.

This moderating pathway is grounded in intergroup contact theory, which suggests that the quality of contact is more directly associated with learners’ emotional responses to the target language community, such as belonging, trust, and interpersonal closeness, than with subsequent behavioural intentions. In this regard, cross-cultural contact quality is expected to shape whether learners perceive themselves as accepted members of the target language community, thereby influencing the association between identity negotiation and sense of belonging.

Based on the above theoretical framework, the following hypotheses are proposed:

*H1*: Second language identity negotiation is positively associated with willingness to communicate.

*H2*: Second language identity negotiation is positively associated with a sense of belonging.

*H3*: Sense of belonging mediates the association between second language identity negotiation and willingness to communicate.

*H4*: Cross-cultural contact quality moderates the association between second language identity negotiation and sense of belonging, such that this positive association is stronger among learners reporting higher levels of cross-cultural contact quality.

## 3 Research methods

### 3.1 Participants

This study employed a cluster sampling strategy. Between September and December 2025, undergraduate students majoring in Spanish were recruited from six Chinese universities offering Spanish language programmes in eastern, northern, and southern China. Classes served as the sampling units. A total of 520 questionnaires were distributed. After excluding questionnaires with excessively short completion times, repetitive response patterns, or substantial missing data, 468 valid responses were retained for analysis, yielding a valid response rate of 90.0%.

Among the participants, 346 were female (73.9%) and 122 were male (26.1%), reflecting the predominance of female students in Spanish language programmes in China. Participants ranged in age from 18 to 24 years (*M* = 20.421, SD = 1.511). The sample included 112 first-year students (23.9%), 126 s-year students (26.9%), 130 third-year students (27.8%), and 100 fourth-year students (21.4%). The duration of Spanish language study ranged from one to 4 years (*M* = 2.325, SD = 0.852). In addition, 196 participants (41.9%) reported previous face-to-face or online contact with native Spanish speakers. This study was reviewed and approved by the Ethics Committee of the School of Foreign Languages, Henan University of Science and Technology (Approval No. HAUST-FLS-2025-0910).

### 3.2 Research procedure

Approval to conduct the study was obtained from the relevant departments and teaching staff at each participating university. Questionnaires were administered either in paper format during class or online through the Wenjuanxing platform. Before completing the questionnaire, participants were informed of the purpose of the study, the voluntary nature of participation, and the procedures used to ensure anonymity. Informed consent was obtained from all participants prior to data collection.

The questionnaire employed a five-point Likert scale, and completion required approximately 15 min. To reduce the potential influence of common method bias, several procedural measures recommended by Podsakoff et al. (2003) were adopted. These included anonymous data collection, randomised item order, the inclusion of reverse-coded items, and informing participants that there were no right or wrong answers.

### 3.3 Measurement instruments

All measurement instruments were originally developed in English and translated into Chinese using the back-translation procedure. To ensure semantic equivalence, the translated versions were independently reviewed and refined by two lecturers specialising in Spanish. During the adaptation process, particular attention was paid to ensuring that the wording of each item was linguistically accurate and culturally appropriate for Chinese learners of Spanish. Minor modifications were made to improve clarity and contextual relevance while preserving the conceptual meanings of the original instruments. The final Chinese version was carefully reviewed by the research team prior to formal data collection to ensure the appropriateness of the adapted measures for the target population.

1. *Second language identity negotiation*. Second language identity negotiation was measured using a six-item scale adapted from the identity investment orientation measures developed by Papi and Hiver (2020) and Gao (2021). The scale assesses the extent to which learners actively construct, negotiate, and integrate their second language identity while using Spanish. Sample items include “When speaking Spanish, I actively express aspects of myself that differ from my Chinese self” and “I strive to have my identity recognised by the Spanish-speaking community.” Responses were recorded on a five-point Likert scale ranging from 1 (strongly disagree) to 5 (strongly agree), with higher scores indicating higher levels of second language identity negotiation. In the present study, Cronbach’s α was 0.915.
2. *Sense of belonging*. Sense of belonging was assessed using a five-item version of the Psychological Sense of School Membership (PSSM) scale developed by Goodenow (1993) and adapted for second language learning contexts. The scale measures learners’ perceived acceptance and belonging within the Spanish learning community. A sample item is “I feel that I am a member of the Spanish learning group.” Responses were rated on a five-point Likert scale, with higher scores indicating a stronger sense of belonging. Cronbach’s α for this scale was 0.873.
3. *Willingness to communicate*. Willingness to communicate was measured using the classroom communication subscale of the WTC instrument developed by MacIntyre et al. (1998), based on the revised version proposed by Peng and Woodrow (2010). The six-item scale assesses learners’ willingness to communicate in Spanish during classroom learning activities. A sample item is “I am willing to answer the teacher’s questions in Spanish during class.” Responses were rated on a five-point Likert scale, with higher scores indicating greater willingness to communicate. Cronbach’s α was 0.943.
4. *Cross-cultural contact quality*. Cross-cultural contact quality was measured using a five-item scale adapted from the instruments developed by Vezzali et al. (2015) and Peng (2015). The scale assesses the extent to which learners experience authentic, positive, equal, and close interactions with native Spanish speakers and Spanish-speaking communities. A sample item is “My contact with native Spanish speakers is equal and friendly.” Responses were recorded on a five-point Likert scale, with higher scores indicating higher levels of cross-cultural contact quality. Cronbach’s *α* for this scale was 0.910.

### 3.4 Data analysis strategy

Data analyses were conducted using SPSS 27.0, and Hayes’ PROCESS Macro. First, confirmatory factor analysis (CFA) was performed using AMOS 26.0 to examine the discriminant validity of the measurement model by comparing several competing measurement models. Subsequently, descriptive statistics and Pearson correlation analyses were conducted using SPSS 27.0 to examine the distributions of the study variables and their bivariate relationships. Common method bias was assessed using Harman’s one-factor test.

The moderated mediation model was tested using PROCESS Macro Model 7 (Hayes, 2017). Bootstrap confidence intervals were estimated based on 5,000 resamples. Before the analyses, all continuous variables were standardised (Z-scores). The regression analyses controlled for gender, age, year of study, duration of Spanish study, and previous cross-cultural contact experience.

## 4 Results

### 4.1 Measurement model

To examine the discriminant validity of the measurement model, confirmatory factor analyses were conducted by comparing five competing models. The results showed that the four-factor model, comprising second language identity negotiation, sense of belonging, willingness to communicate, and cross-cultural contact quality as distinct latent constructs, demonstrated the best overall model fit (*χ^2^*/df = 2.47, CFI = 0.946, TLI = 0.938, RMSEA = 0.056, SRMR = 0.043). All fit indices met the recommended criteria proposed by Hu and Bentler (1999). Compared with the alternative three-factor, two-factor, and one-factor models, the four-factor model showed a better fit to the data (see Table 1), supporting the discriminant validity of the four study constructs.

**Table 1:** Comparison of confirmatory factor analysis model fit indices.

Model	*χ^2^*/df	CFI	TLI	RMSEA	SRMR
Four-factor model	2.470	0.946	0.938	0.056	0.043
Three-factor modelᵃ	2.850	0.883	0.869	0.081	0.066
Three-factor modelᵇ	3.020	0.867	0.851	0.086	0.070
Two-factor model	3.940	0.774	0.752	0.112	0.091
One-factor model	6.580	0.561	0.521	0.168	0.128

ᵃ Second language identity negotiation and sense of belonging were combined into a single factor. ᵇ Sense of belonging and willingness to communicate were combined into a single factor. *N* = 468.

### 4.2 Descriptive statistics and correlation analysis

Descriptive statistics and Pearson correlation coefficients for the study variables are presented in Table 2. Second language identity negotiation, sense of belonging, willingness to communicate, and cross-cultural contact quality were all positively correlated with one another, and all correlation coefficients were statistically significant (*p* < 0.01).

**Table 2:** Descriptive statistics and correlations among the study variables.

Variable	1	2	3	4
1. Second language identity negotiation	1.000			
2. Sense of belonging	0.396^**^	1.000		
3. Willingness to communicate	0.394^**^	0.495^**^	1.000	
4. Cross-cultural contact quality	0.389^**^	0.326^**^	0.227^**^	1.000
M	3.676	3.703	3.240	3.546
SD	0.606	0.568	0.742	0.647

*p* < 0.01. *N = 468.* **M = mean; SD = standard deviation. *p* < 0.01. Correlation is significant at the 0.01 level (2-tailed). *N* = 468.

### 4.3 Common method Bias test

Common method bias was assessed using Harman’s one-factor test. All measurement items were entered into an exploratory factor analysis. The first unrotated factor accounted for 28.74% of the total variance, which was below the recommended threshold of 40% (Podsakoff et al., 2003). In addition, the one-factor model reported in Table 1 showed substantially poorer model fit than the four-factor model (ΔCFI = 0.385). These findings suggest that common method bias was unlikely to pose a serious concern in the present study.

### 4.4 Hypothesis testing

The results of the hierarchical regression analyses are presented in Tables 2–5. After controlling for gender, age, year of study, duration of Spanish study, and previous cross-cultural contact experience, second language identity negotiation was positively associated with willingness to communicate (*β* = 0.396, *t* = 9.302, *p* < 0.001), supporting H1. In addition, second language identity negotiation was positively associated with sense of belonging (*β* = 0.395, *t* = 9.268, *p* < 0.001), supporting H2.

When sense of belonging was entered into the regression model, it was positively associated with willingness to communicate (*β* = 0.398, *t* = 9.312, *p* < 0.001). The regression coefficient for second language identity negotiation remained statistically significant but was reduced (*β* = 0.239, *t* = 5.610, *p* < 0.001), indicating a pattern consistent with partial mediation.

The indirect effect was further examined using a bootstrap procedure with 5,000 resamples. The indirect effect of second language identity negotiation on willingness to communicate through sense of belonging was 0.157 (SE = 0.024), and the 95% confidence interval did not include zero, indicating that the indirect effect was statistically significant. Therefore, H3 was supported. The indirect effect accounted for 39.7% of the total effect.

Among the control variables, gender, age, year of study, duration of Spanish study, and previous cross-cultural contact experience did not show statistically significant associations with either sense of belonging or willingness to communicate. Therefore, these variables are not discussed further.

### 4.5 Moderated mediation analysis

The moderated mediation model was examined using PROCESS Macro Model 7 (Hayes, 2017), with second language identity negotiation as the independent variable, sense of belonging as the mediator, willingness to communicate as the dependent variable, and cross-cultural contact quality as the moderator. Gender, age, year of study, duration of Spanish study, and previous cross-cultural contact experience were included as control variables. Bootstrap confidence intervals were estimated based on 5,000 resamples. The results are presented in Table 3.

**Table 3:** Regression results for the moderated mediation model (process model 7).

Variable	*b*	SE	*t*	*p*	*R* ^2^	*F*
Dependent variable: sense of belonging					0.237	17.865^***^
Second language identity negotiation	0.312	0.044	7.044	<0.001		
Cross-cultural contact quality	0.198	0.044	4.465	<0.001		
Second language identity negotiation × Cross-cultural contact quality	0.201	0.043	4.684	<0.001		
Dependent variable: willingness to communicate					0.299	27.989^***^
Sense of belonging	0.398	0.043	9.312	<0.001		
Second language identity negotiation	0.239	0.043	5.610	<0.001		

^**^p < 0.001. *** indicates statistical significance at the 0.001 level. Coefficients are based on standardised (z-score) variables. Control variables included gender, age, year of study, duration of Spanish study, and previous cross-cultural contact experience. *N* = 468.

For the model predicting sense of belonging, second language identity negotiation (*b* = 0.312, *t* = 7.044, *p* < 0.001) and cross-cultural contact quality (*b* = 0.198, *t* = 4.465, *p* < 0.001) were both positively associated with sense of belonging. In addition, the interaction between second language identity negotiation and cross-cultural contact quality was statistically significant (*b* = 0.201, *t* = 4.684, *p* < 0.001), indicating that cross-cultural contact quality moderated the association between second language identity negotiation and sense of belonging. For the model predicting willingness to communicate, sense of belonging was positively associated with willingness to communicate (*b* = 0.398, *t* = 9.312, *p* < 0.001), and the coefficient for second language identity negotiation remained statistically significant (*b* = 0.239, *t* = 5.610, *p* < 0.001).

The index of moderated mediation was 0.080 (SE = 0.018), and the 95% bootstrap confidence interval ranged from 0.047 to 0.116. Because the confidence interval did not include zero, the moderated mediation effect was considered statistically significant. Conditional indirect effects were further estimated at three levels of cross-cultural contact quality, corresponding to one standard deviation below the mean, the mean, and one standard deviation above the mean (see Table 4). The indirect effect increased as cross-cultural contact quality increased, with estimates of 0.044 (95% CI = [−0.005, 0.095]), 0.124 (95% CI = [0.085, 0.168]), and 0.204 (95% CI = [0.149, 0.264]) at the low, moderate, and high levels, respectively.

**Table 4:** Conditional indirect effects at different levels of cross-cultural contact quality.

Cross-cultural contact quality	Indirect effect	Boot SE	95% Boot CI (Lower)	95% Boot CI (Upper)
Low (−1 SD)	0.044	0.025	−0.005	0.095
Mean	0.124	0.021	0.085	0.168
High (+1 SD)	0.204	0.030	0.149	0.264

Bootstrap confidence intervals were estimated based on 5,000 resamples. CI = confidence interval. The indirect effect was not statistically significant at the low level of cross-cultural contact quality because the 95% bootstrap confidence interval included zero.

The indirect effect was not statistically significant at the low level of cross-cultural contact quality, as the confidence interval included zero. In contrast, the indirect effects at the moderate and high levels were statistically significant.

A simple slope analysis was conducted to further examine the moderating effect of cross-cultural contact quality (see Table 5). When cross-cultural contact quality was low (−1 SD), the association between second language identity negotiation and sense of belonging was not statistically significant (*b* = 0.111, SE = 0.062, *t* = 1.792, *p* = 0.074). In contrast, when cross-cultural contact quality was high (+1 SD), the association was statistically significant and substantially stronger (*b* = 0.513, SE = 0.061, *t* = 8.379, *p* < 0.001). These findings indicate that higher levels of cross-cultural contact quality strengthened the positive association between second language identity negotiation and sense of belonging, thereby supporting H4 (Figure 1).

**Table 5:** Simple slope analysis of the association between second language identity.

Cross-cultural contact quality	*b*	SE	*t*	*p*
Low (−1 SD)	0.111	0.062	1.792	0.074
Mean	0.312	0.044	7.044	<0.001
High (+1 SD)	0.513	0.061	8.379	<0.001

Negotiation and sense of belonging at different levels of cross-cultural contact quality.

**Figure 1:**
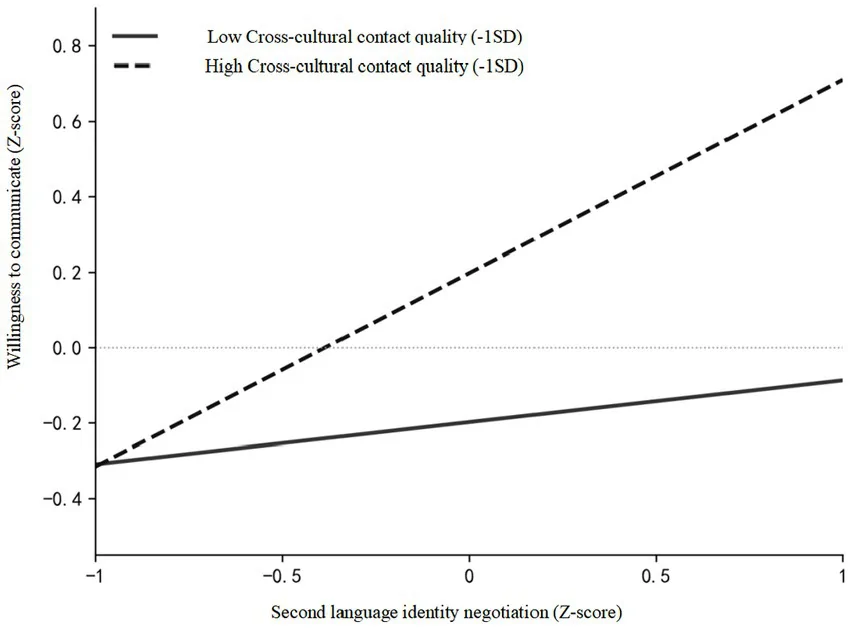
Simple slope plot of the association between second language identity negotiation and sense of belonging at different levels of cross-cultural contact quality. The x-axis represents standardized scores for second language identity negotiation, and the y-axis represents standardized scores for sense of belonging. Low and high levels of cross-cultural contact quality are defined as one standard deviation below and above the mean, respectively. The dependent variable is sense of belonging. *N* = 468.

## 5 Discussion

### 5.1 Key findings and interpretation

The present study examined a moderated mediation model to investigate the relationships among second language identity negotiation, sense of belonging, willingness to communicate (WTC), and cross-cultural contact quality among Chinese learners of Spanish. Overall, the findings supported all four hypotheses and provided further evidence for the psychological and contextual processes associated with learners’ willingness to communicate.

Although the proposed moderated mediation model was theoretically derived, the cross-sectional nature of the present study means that the findings should be interpreted as patterns of association that are consistent with, rather than definitive evidence for, the proposed directional relationships.

First, the findings showed that second language identity negotiation was positively associated with willingness to communicate, supporting H1. This finding is consistent with identity theory, which conceptualises language learning as an ongoing process of identity investment and negotiation (Norton, 2013; Darvin and Norton, 2015). Learners who perceive greater integration between their second language identity and their sense of self may be more willing to use the target language for communication because language use becomes a means of expressing their identities rather than simply completing learning tasks. This interpretation is also consistent with the findings of Papi and Hiver (2020), who highlighted the importance of identity orientation in second language learning. For Chinese learners of Spanish, whose target language differs substantially from Chinese in both linguistic and cultural characteristics, negotiating a second language identity may represent a particularly important aspect of their learning experience, which may help explain its close association with willingness to communicate.

Second, the findings indicated that second language identity negotiation was positively associated with sense of belonging, and that sense of belonging mediated the association between second language identity negotiation and willingness to communicate, supporting H2 and H3. This pattern is consistent with belonging theory (Baumeister and Leary, 1995) and the WTC framework proposed by MacIntyre et al. (1998). Learners who perceive greater acceptance within the target language community are more likely to develop a stronger sense of belonging, which, in turn, is associated with greater willingness to communicate. The indirect effect accounted for approximately 40% of the total effect, suggesting that sense of belonging represents an important pathway linking identity negotiation and WTC. At the same time, the direct association between second language identity negotiation and willingness to communicate remained statistically significant, suggesting that sense of belonging represents an important but not exclusive psychological mechanism underlying this relationship. This finding is consistent with MacIntyre et al.'s (1998) multi-level model, which proposes that willingness to communicate is jointly influenced by multiple affective, motivational, and social factors. Beyond fostering a sense of belonging, may also be associated with higher levels of learners’ ideal L2 self, communication confidence, learning motivation, or perceived communicative competence, all of which have been identified as important antecedents of WTC. Therefore, the present findings suggest that identity negotiation may influence willingness to communicate through multiple psychological pathways rather than through belonging alone (Cao, 2014; Khajavy et al., 2016; Zhang M. et al., 2026).

Although the proposed model explained a meaningful proportion of the variance in sense of belonging (23.7%) and willingness to communicate (29.9%), a substantial proportion of variance remained unexplained. This finding suggests that willingness to communicate is a multifaceted construct that is likely to be associated with additional psychological, social, and contextual factors beyond those examined in the present study. Future research may therefore consider incorporating other theoretically relevant variables, such as language anxiety, intercultural communication confidence, or motivational orientations, to provide a more comprehensive understanding of learners’ willingness to communicate.

The present study also found that cross-cultural contact quality moderated the association between second language identity negotiation and sense of belonging, supporting H4. Specifically, the positive association between second language identity negotiation and sense of belonging was stronger among learners reporting higher levels of cross-cultural contact quality. This finding is consistent with intergroup contact theory, which proposes that positive and meaningful interactions promote favourable emotional responses toward members of other groups (Allport, 1954; Pettigrew and Tropp, 2006). Authentic, equal, and supportive interactions with native Spanish speakers may provide opportunities for learners to validate and reinforce their second language identities, thereby strengthening their sense of belonging within the target language community.

By contrast, when cross-cultural contact quality is relatively low, learners may have fewer opportunities to transform their negotiated second language identities into genuine feelings of membership within the target language community. Low-quality intercultural interactions may be characterised by limited reciprocity, superficial communication, or even experiences of misunderstanding and social distance. Under such conditions, learners’ internally negotiated identities are less likely to receive social validation from members of the target language community. Consequently, identity negotiation alone may be insufficient to generate a strong sense of belonging. This finding highlights that belonging is not solely an individual psychological achievement but also depends on supportive interpersonal and sociocultural environments that enable learners to experience acceptance and recognition.

The findings also support the decision to position cross-cultural contact quality as a moderator of the first stage of the mediation model. From the perspective of intergroup contact theory, contact is expected to be more closely related to learners’ emotional experiences than to subsequent behavioural intentions. Consistent with this expectation, the conditional indirect effect increased as cross-cultural contact quality increased, suggesting that meaningful cross-cultural experiences are particularly relevant to the association between identity negotiation and emotional belonging. This interpretation is broadly consistent with previous studies highlighting the relationship between contact experiences and positive emotions in language learning (Dewaele and MacIntyre, 2014; Dewaele et al., 2024; Dewaele and Saito, 2024). Taken together, these findings further support intergroup contact theory by demonstrating that contextual characteristics of intercultural interactions shape learners’ emotional adaptation before influencing subsequent communicative behaviour.

### 5.2 Theoretical contributions

First, this study extends previous research on willingness to communicate by integrating identity theory, belonging theory, and MacIntyre et al.'s (1998) WTC framework into a unified explanatory model. Rather than treating identity negotiation and willingness to communicate as two independent research streams, the present study demonstrates that learners’ sense of belonging functions as a key affective mechanism linking identity negotiation with communicative intentions. This integrated perspective enriches current understanding of how identity-related processes are translated into communication behaviour in second language learning.

Second, this study used intergroup contact theory to point to the significance of contact quality in the crosscultural context as a contextual factor. This adds to the existing literature by showing that identity-related processes and emotional outcomes can be differently related in different social contexts.

Third, this study concentrates on a learner group, namely Chinese students learning Spanish, that has been relatively underresearched in previous studies; thus it expands the empirical literature on “languages other than English” (LOTEs). The results also offer further support to the studies of the applicability of identity theory, belonging theory, and WTC framework in other cultures and languages.

### 5.3 Practical implications

This study offers several practical implications for Spanish language teaching and learning.

First, the finding that second language identity negotiation was positively associated with both learners’ sense of belonging and willingness to communicate suggests that language instruction should extend beyond the development of linguistic knowledge and communicative skills to include opportunities for identity development. Teachers are encouraged to design activities that help learners actively explore, express, and negotiate their emerging second language identities. For example, reflective journals, autobiographical writing, role-playing, intercultural dialogue, and discussions of multicultural texts can encourage learners to connect language learning with their personal experiences and future aspirations. Such activities may facilitate learners’ identification with the Spanish-speaking community, thereby strengthening their sense of belonging and ultimately promoting their willingness to communicate.

Second, because sense of belonging emerged as a significant mediator, fostering a supportive and inclusive classroom environment should be regarded as a central pedagogical objective rather than merely a desirable classroom characteristic. Teachers should intentionally create learning environments in which students feel respected, accepted, and encouraged to participate without fear of negative evaluation. Recent research has further shown that timely and supportive teacher feedback in technology-enhanced learning environments can strengthen learners’ motivation and self-efficacy, thereby promoting active engagement in language learning (Zheng and Pan, 2026). Although the present study focused on face-to-face language learning, similar supportive feedback practices may also contribute to learners’ sense of belonging and willingness to communicate in blended and online learning environments.

Third, the moderating effect of cross-cultural contact quality indicates that simply increasing opportunities for intercultural contact may not be sufficient. Instead, educational institutions should prioritise meaningful, reciprocal, and sustained interactions with native Spanish speakers and Spanish-speaking communities. Such opportunities may be provided through language partner programmes, virtual exchanges, collaborative online international learning (COIL), study-abroad programmes, community engagement projects, and culturally focused workshops. More importantly, educators should pay particular attention to the quality of these interactions by encouraging mutual respect, authentic communication, equal participation, and sustained engagement, as these conditions appear to facilitate the transformation of learners’ negotiated identities into a stronger sense of belonging.

### 5.4 Limitations and future research

First, the cross-sectional design does not permit causal inferences. Future research could employ longitudinal or experimental designs to examine the temporal relationships among the study variables.

Second, all data were collected using self-report questionnaires. Although the results suggested that common method bias was unlikely to pose a serious concern, self-report data remain subject to potential response bias. Future studies could incorporate multiple data sources, such as teacher ratings, peer evaluations, or observations of learners’ actual communicative behaviour.

Third, the sample consisted exclusively of undergraduate students majoring in Spanish at Chinese universities. Accordingly, caution is warranted when generalising the findings to other learner populations, such as adult learners or individuals studying Spanish in informal learning settings.

Fourth, this study examined cross-cultural contact quality only as a moderator of the association between second language identity negotiation and sense of belonging. Future research could investigate whether cross-cultural contact quality also moderates the association between sense of belonging and willingness to communicate, or whether other contextual or individual variables, such as language anxiety or motivational orientation, function as moderators within the proposed model.

Finally, future research could adopt person-centred approaches, such as latent profile analysis, to identify distinct learner profiles based on second language identity negotiation and cross-cultural contact quality. Such approaches may provide a more nuanced understanding of individual differences in the psychological processes associated with willingness to communicate. In addition, with the rapid development of AI-supported language learning environments, virtual exchange programmes, and intelligent conversational agents, future research could investigate whether technology-mediated intercultural interactions facilitate learners’ identity negotiation, strengthen their sense of belonging, and ultimately enhance their willingness to communicate. Recent studies have highlighted the growing importance of learners’ emotional engagement, technology trust, and self-efficacy in digitally mediated language learning environments (Liu et al., 2026; Zhang Q. et al., 2026; Zheng and Pan, 2026). Extending the present theoretical framework to technology-enhanced language learning contexts would therefore represent a promising direction for future research.

## 6 Conclusion

The present study examined the relationships among second language identity negotiation, sense of belonging, willingness to communicate, and cross-cultural contact quality among Chinese learners of Spanish using a moderated mediation model. The findings showed that: (1) second language identity negotiation was positively associated with both willingness to communicate and sense of belonging; (2) sense of belonging mediated the association between second language identity negotiation and willingness to communicate; and (3) cross-cultural contact quality moderated the association between second language identity negotiation and sense of belonging, such that this association was stronger among learners reporting higher levels of cross-cultural contact quality.

Overall, these findings contribute to a better understanding of the psychological and contextual processes associated with willingness to communicate in second language learning. They also provide empirical evidence that may inform identity-oriented pedagogy and the design of meaningful cross-cultural learning experiences in Spanish language education.

## Data Availability

The raw data supporting the conclusions of this article will be made available by the authors, without undue reservation.

## References

[ref1] Allport G. W. (1954). The Nature of Prejudice. Cambridge, MA, USA: Addison-Wesley.

[ref2] Baumeister R. F. Leary M. R. (1995). The need to belong: desire for interpersonal attachments as a fundamental human motivation. Psychol. Bull. 117, 497–529. doi: 10.1037/0033-2909.117.3.497, 7777651

[ref3] Block D. (2007). The rise of identity in SLA research, post firth and Wagner (1997). Mod. Lang. J. 91, 863–876. doi: 10.1111/j.1540-4781.2007.00674.x

[ref4] Botes E. Dewaele J.-M. Greiff S. (2022). Taking stock: a meta-analysis of the effects of foreign language enjoyment. Stud. Second Lang. Learn. Teach. 12, 205–232. doi: 10.14746/ssllt.2022.12.2.3

[ref5] Cao Y. (2014). A sociocognitive perspective on second language classroom willingness to communicate. TESOL Q. 48, 789–814. doi: 10.1002/tesq.155

[ref6] Darvin R. Norton B. (2015). Identity and a model of investment in applied linguistics. Annu. Rev. Appl. Linguist. 35, 36–56. doi: 10.1017/S0267190514000191

[ref8] Dewaele J.-M. MacIntyre P. D. (2014). The two faces of Janus? Anxiety and enjoyment in the foreign language classroom. Stud. Second Lang. Learn. Teach. 4, 237–274. doi: 10.14746/ssllt.2014.4.2.5

[ref9001] Dewaele J.-M. Saito K. (2024). Are enjoyment, anxiety and attitudes/motivation different in English foreign language classes compared to LOTE classes? Stud. Second Lang. Learn. Teach. 14, 171–191. doi: 10.14746/ssllt.42376

[ref7] Dewaele J.-M. Guedat-Bittighoffer D. Botes E. Dat M.-A. (2024). How intense and authentic communication in foreign language classes has a positive effect on learner emotions. Can. Mod. Lang. Rev. 80, 262–284. doi: 10.3138/cmlr-2023-0012

[ref9] Dörnyei Z. (2005). The Psychology of the Language Learner: Individual Differences in Second Language Acquisition. Mahwah, NJ, USA: Lawrence Erlbaum.

[ref10] Dörnyei Z. (2009). “The L2 motivational self system,” in Motivation, Language Identity and the L2 Self, eds. Dörnyei Z. Ushioda E. (Bristol, UK: Multilingual Matters).

[ref9002] Gao F. (2021). Negotiation of native linguistic ideology and cultural identities in English learning: A cultural schema perspective. J. Multiling. Multicult. Dev. 42, 551–564. doi: 10.1080/01434632.2020.1857389

[ref11] Gao X. Jia Z. Zhou Y. (2015). EFL learning and identity development: a longitudinal study in 5 universities in China. J. Lang. Identity Educ. 14, 137–158. doi: 10.1080/15348458.2015.1041338

[ref12] Goodenow C. (1993). The psychological sense of school membership among adolescents: scale development and educational correlates. Psychol. Sch. 30, 79–90. doi: 10.1002/1520-6807(199301)30:1<>3.0.CO;2-X

[ref13] Hayes A. F. (2017). Introduction to Mediation, Moderation, and Conditional process Analysis: A Regression-Based Approach. 2nd Edn. New York, NY, USA: Guilford Press.

[ref14] Hu L. Bentler P. M. (1999). Cutoff criteria for fit indexes in covariance structure analysis: conventional criteria versus new alternatives. Struct. Equ. Model. 6, 1–55. doi: 10.1080/10705519909540118

[ref15] Kang S.-J. (2005). Dynamic emergence of situational willingness to communicate in a second language. System 33, 277–292. doi: 10.1016/j.system.2004.10.004

[ref16] Khajavy G. H. Ghonsooly B. Hosseini Fatemi A. Choi C. W. (2016). Willingness to communicate in English: a microsystem model in the Iranian EFL classroom context. TESOL Q. 50, 154–180. doi: 10.1002/tesq.204

[ref17] Kormos J. Csizér K. (2014). The interaction of motivation, self-regulatory strategies, and autonomous learning behavior in different learner groups. TESOL Q. 48, 275–299. doi: 10.1002/tesq.129

[ref18] Lee J. S. Lu Y. (2023). L2 motivational self system and willingness to communicate in the classroom and extramural digital contexts. Comput. Assist. Lang. Learn. 36, 126–148. doi: 10.1080/09588221.2021.1901746

[ref19] Liu H. Fan J. Pan X. (2026). Exploring the complex interplay of supportive factors, learners' emotions on their sustained use of AI-enhanced learning technologies: insights from the combined SEM and fsQCA approaches. System 141:104109. doi: 10.1016/j.system.2026.104109

[ref20] Luo C. García Parejo I. (2022). Estudio correlacional entre la identidad y la motivación de los estudiantes chinos en el aprendizaje del español como lengua extranjera. RLA., Rev. Lingüíst. Teór. Apl. 60, 15–38. doi: 10.29393/RLA60-1ECCI20001

[ref22] MacIntyre P. D. Legatto J. J. (2011). A dynamic system approach to willingness to communicate: developing an idiodynamic method to capture rapidly changing affect. Appl. Linguist. 32, 149–171. doi: 10.1093/applin/amq037

[ref21] MacIntyre P. D. Clément R. Dörnyei Z. Noels K. A. (1998). Conceptualizing willingness to communicate in a L2: a situational model of L2 confidence and affiliation. Mod. Lang. J. 82, 545–562. doi: 10.1111/j.1540-4781.1998.tb05543.x

[ref23] McCroskey J. C. Baer J. E. (1985). “Willingness to communicate: the construct and its measurement,” in Paper Presented at the Annual Convention of the Speech Communication Association, (Denver).

[ref24] Noels K. A. Lascano D. I. V. Saumure K. (2019). The development of self-determination across the language course. Stud. Second. Lang. Acquis. 41, 821–851. doi: 10.1017/S0272263118000189

[ref25] Norton B. (2000). Identity and Language Learning: Gender, Ethnicity and Educational Change. Harlow, UK: Longman.

[ref26] Norton B. (2013). Identity and Language Learning: Extending the Conversation. 2nd Edn. Bristol, UK: Multilingual Matters.

[ref27] Papi M. Hiver P. (2020). Language learning motivation as a complex dynamic system: a global perspective of truth, control, and value. Mod. Lang. J. 104, 209–232. doi: 10.1111/modl.12624

[ref28] Pawlak M. Mystkowska-Wiertelak A. (2015). Investigating the dynamic nature of L2 willingness to communicate. System 50, 1–9. doi: 10.1016/j.system.2015.02.001

[ref29] Peng J.-E. (2015). L2 motivational self system, attitudes, and affect as predictors of L2 WTC: an imagined community perspective. Asia Pac. Educ. Res. 24, 433–443. doi: 10.1007/s40299-014-0195-0

[ref30] Peng J.-E. (2022). Willingness to communicate in an L2 and integrative motivation among Chinese learners of English: a mixed-methods study. System 108:102802. doi: 10.1016/j.system.2022.102802

[ref31] Peng J.-E. Woodrow L. (2010). Willingness to communicate in English: a model in the Chinese EFL classroom context. Lang. Learn. 60, 834–876. doi: 10.1111/j.1467-9922.2010.00576.x

[ref32] Peng J.-E. Wu L. (2022). Motivational profiles of Chinese university students majoring in Spanish: a comparative study. J. Multiling. Multicult. Dev. 45, 1938–1955. doi: 10.1080/01434632.2022.2035740

[ref33] Pettigrew T. F. (1998). Intergroup contact theory. Annu. Rev. Psychol. 49, 65–85. doi: 10.1146/annurev.psych.49.1.65, 15012467

[ref34] Pettigrew T. F. Tropp L. R. (2006). A meta-analytic test of intergroup contact theory. J. Pers. Soc. Psychol. 90, 751–783. doi: 10.1037/0022-3514.90.5.751, 16737372

[ref35] Podsakoff P. M. MacKenzie S. B. Lee J.-Y. Podsakoff N. P. (2003). Common method biases in behavioral research: a critical review of the literature and recommended remedies. J. Appl. Psychol. 88, 879–903. doi: 10.1037/0021-9010.88.5.879, 14516251

[ref36] Ryan R. M. Deci E. L. (2000). Self-determination theory and the facilitation of intrinsic motivation, social development, and wellbeing. Am. Psychol. 55, 68–78. doi: 10.1037/0003-066X.55.1.68, 11392867

[ref37] Tang F. Calafato R. (2022a). “The culture of China is broad and profound, with all rivers flowing into the sea”: plurilingual and pluricultural competence and identity among university students in China. J. Multiling. Multicult. Dev. doi: 10.1080/01434632.2022.2083625

[ref39] Tsang A. (2022). Examining the relationship between language and cross-cultural encounters: avenues for promoting intercultural interaction. J. Multiling. Multicult. Dev. 43, 98–110. doi: 10.1080/01434632.2020.1725526

[ref40] Ushioda E. (2011). Language learning motivation, self and identity: current theoretical perspectives. Comput. Assist. Lang. Learn. 24, 199–210. doi: 10.1080/09588221.2010.538701

[ref41] Vezzali L. Hewstone M. Capozza D. Giovannini D. Wölfer R. (2015). Improving intergroup relations with extended and vicarious forms of indirect contact. Eur. Rev. Soc. Psychol. 25, 314–389. doi: 10.1080/10463283.2014.982948, 37339054

[ref42] Walton G. M. Cohen G. L. (2007). A question of belonging: race, social fit, and achievement. J. Pers. Soc. Psychol. 92, 82–96. doi: 10.1037/0022-3514.92.1.82, 17201544

[ref43] Wei X. Xu Q. (2022). Predictors of willingness to communicate in a second language (L2 WTC): toward an integrated L2 WTC model from the socio-psychological perspective. Foreign Lang. Ann. 55, 258–282. doi: 10.1111/flan.12595

[ref44] Wen W. P. Clément R. (2003). A Chinese conceptualisation of willingness to communicate in ESL. Lang. Cult. Curric. 16, 18–38. doi: 10.1080/07908310308666654

[ref45] Yashima T. (2002). Willingness to communicate in a second language: the Japanese EFL context. Mod. Lang. J. 86, 54–66. doi: 10.1111/1540-4781.00136

[ref46] Yashima T. Zenuk-Nishide L. Shimizu K. (2004). The influence of attitudes and affect on willingness to communicate and second language communication. Lang. Learn. 54, 119–152. doi: 10.1111/j.1467-9922.2004.00250.x

[ref47] Zarrinabadi N. (2014). Communicating in a second language: investigating the effect of teacher on learners’ willingness to communicate. System 42, 288–295. doi: 10.1016/j.system.2013.12.014

[ref48] Zhang J. Beckmann N. Beckmann J. F. (2018). To talk or not to talk: a review of situational antecedents of willingness to communicate in the second language classroom. System 72, 226–239. doi: 10.1016/j.system.2018.01.003

[ref49] Zhang M. Liu L. Pan X. (2026). Examining the association between digital technology literacy and technology-based teaching design: the mediating effect of technology trust and technological self-efficacy. Front. Psychol. 17:1858642. doi: 10.3389/fpsyg.2026.1858642, 42266775 PMC13243424

[ref50] Zheng Q. Pan J. (2026). Examining the impact of online teacher feedback on learningengagement: the mediating roles of technological self-efficacy and learning motivation. Interact. Learn. Environ. 34,2560–2573. doi: 10.1080/10494820.2025.2547263

